# Production of IL-31 in CD45RO^+^CLA^+^H4R^+^ T Cells in Atopic Dermatitis

**DOI:** 10.3390/jcm10091976

**Published:** 2021-05-04

**Authors:** Chul Hwan Bang, Ji Young Song, Yu Mee Song, Ji Hyun Lee, Young Min Park, Jun Young Lee

**Affiliations:** 1 Department of Dermatology, Seoul St. Mary’s Hospital, College of Medicine, The Catholic University of Korea, 222, Banpo-daero, Seocho-gu, Seoul 06591, Korea; mrbangga@catholic.ac.kr (C.H.B.); yumeesong@naver.com (Y.M.S.); ejee@catholic.ac.kr (J.H.L.); 96015367@cmcnu.or.kr (Y.M.P.); 2 Program of Immunology & Microbiology, Department of Biomedicine & Health Science, Graduate School, The Catholic University of Korea, 222, Banpo-daero, Seocho-gu, Seoul 06591, Korea; sjy8699@gmail.com

**Keywords:** Interleukin-31, histamine-4-receptor, atopic dermatitis

## Abstract

IL-31 is involved in pruritus in atopic dermatitis (AD) and the pathogenesis of AD. However, the mechanism of IL-31 production is not fully understood. We sought to investigate the association between CD45RO^+^CLA^+^H4R^+^ T cells and IL-31 production. Immunofluorescence studies were performed retrospectively on punch-biopsy specimens from five people with AD and three healthy controls. In addition, blood samples were collected prospectively from eight patients with AD and eight healthy controls for sorting CD45RO^+^CLA^+^H4R^+^ T cells. There was no overlap of patients between the biopsy group and the blood sampling group. Sorted cells were stimulated with 4-methylhistamine (4MH), and the level of IL-31 was measured by an enzyme-linked immunosorbent assay. Immunofluorescence showed co-localization of H4R and IL-31 in lesional AD skin but not in normal skin of healthy controls. The proportion of CLA^+^H4R^+^ T cells among CD3^+^CD45RO^+^ lymphocytes was 18.3 ± 6.2% in patients with AD and 11.2 ± 7.6% in healthy controls. In the AD group, production of IL-31 by CD45RO^+^CLA^+^H4R^+^ T cells increased from 32.4 ± 13.3 pg/mL to 47.5 ± 18.7 pg/mL by 4MH stimulation after 24 h (*p* < 0.001). However, in the control group, production of IL-31 was 20.1 ± 10.6 pg/mL without and 22.1 ± 9.3 pg/mL with 4MH stimulation (*p* > 0.05). According to our study, CD45RO^+^CLA^+^H4R^+^ T cells are an important source of IL-31 in AD, and may be a target for treatment of IL-31-induced pruritus.

## 1. Introduction

Interleukin (IL)-31 is a well-known Th2-cell-derived cytokine involved in promoting skin disorders and regulating allergic diseases such as atopic dermatitis (AD) [1,2]. AD is a common skin disease characterized by chronic relapsing skin inflammation and itching, and the severity of AD is correlated with IL-31. Moreover, IL-31 concentration is correlated with IL-4 and IL-13, which act as key cytokines of AD [2]. In addition, IL-31 is known to cause AD exacerbation by not only directly causing itching, but also by affecting barrier function and dorsal root ganglia or nerve innervation [2]. There were attempts to treat atopic dermatitis by blocking IL-31 or IL-31 receptor. Anti-IL-31 receptor A antibody significantly improves pruritus in patients with moderate-to-severe AD [3,4].

The mRNA and protein expressions of IL-31 are largely restricted to CD4^+^ T cells, particularly skin-homing CD45RO^+^ (memory) cutaneous lymphocyte-associated antigen-positive (CLA^+^) T cells, but not CD45RA^+^ naïve T-cell populations, in people who are healthy and in patients with AD. However, skin samples from patients with AD contain more CLA^+^ T cells than do samples from controls [5]. This suggests that CD45RO^+^CLA^+^ T cells have a key role in the etiology of AD and IL-31 production in AD lesions. IL-31 is also produced by Th1 cells, CD8^+^ T cells, monocytes/macrophages, dendritic cells, keratinocytes, eosinophils, basophils, mast cells, and fibroblasts, but Cevikbas et al. reported that Th2 cells are the major source of IL-31 in human AD [6,7].

IL-31 induces pruritus by direct action in the skin and also by an IL-31 cytokine-triggered neuroimmune circuit that induces itching in patients with Th2-dominated skin diseases by activating IL-31RA on sensory nerves [7]. Thus, blocking the action of IL-31 or IL-31-containing Th2 cells is an important target for controlling pruritus in AD. Although IL-4, IL-33, physical and bacterial stress, staphylococcal enterotoxin B (SEB), and antimicrobial peptides induce IL-31 production, the mechanism of IL-31 secretion in these cells is not fully known [2,6,8,9].

The histamine-4-receptor (H4R) is a recently described histamine receptor that is mainly expressed on several types of hematopoietic cells and is important in histamine-induced activation of inflammatory cells such as mast cells, eosinophils, monocytes, dendritic cells, and T cells [10,11]. H4R mediates proinflammatory functions in allergic inflammation including AD [12]. Gutzmer et al. reported that human CD4^+^ T cells express a functional H4R. H4R is upregulated under Th2 conditions, and H4R stimulation leads to induction of IL-31 in vitro [11]. However, to the best of our knowledge, no investigation has found an association between IL-31 and H4R in human skin and blood samples of patients with AD.

The purpose of this study was to examine the hypothesis that H4R is predominantly located on IL-31-producing T cells, and that activation of H4R leads to IL-31 production in these cells in patients with AD. Since Bilsborough et al. reported that IL-31 is mainly expressed in CD45RO^+^CLA^+^ T cells [5], we investigated the association between CD45RO^+^CLA^+^H4R^+^ T cells and IL-31 production.

## 2. Materials and Methods

### 2.1. Human Tissue Samples

We used biopsy samples of lesional skin from 5 patients with AD retrospectively. As a control, normal skin samples were obtained from three patients who underwent surgery such as lipoma excision. Each tissue sample was stored at −80 °C. This study was approved by the Institutional Review Board of Seoul St. Mary’s Hospital (KC16SISI0619).

### 2.2. Immunofluorescence for Skin Tissue

Immunofluorescence was performed on 5-μm sections of 4% paraformaldehyde-fixed, paraffin wax-embedded tissue. Following dewaxing and rehydration, antigen retrieval was performed using citrate buffer. Sections were blocked with 10% bovine serum albumin (BSA) in phosphate-buffered saline with tween 20 °C (PBST) at room temperature for 1 h. Sections were incubated with polyclonal IgG goat antibody specific to human IL-31 (PA5-47405; Thermo Fisher, Rockford, IL, USA) and polyclonal rabbit antibody specific to human H4R (SP4670P; OriGene Technologies, Rockville, MD, USA) overnight at 4 °C. After rinsing with phosphate-buffered saline (PBS), sections were secondarily stained with Alexa Fluor^®^ 594 chicken anti-rabbit IgG (A-21442; Thermo Fisher) and Alexa Fluor^®^ 488 donkey anti-goat IgG (A-110555; Thermo Fisher) for 2 h in the dark at 37 °C. After washes with PBS, cover slips were mounted with Vectashield Antifade Mounting Medium (Vector Laboratories, Burlingame, CA, USA) containing 4′,6-diamidino-2-phenylindole (DAPI), to counterstain cellular nuclei. Immunofluorescence images were acquired using a fluorescence microscope (Axiovert 200, Zeiss, Germany). We counted the number of localized cells in a high-power field (×200) at a random two sites of biopsy section per patient.

### 2.3. AD Patient Recruitment and Blood Samples

We recruited 8 patients with moderate-to-severe AD and 8 healthy controls from the dermatology clinic of Seoul St. Mary’s Hospital from 1 March 2018 to 31 August 2018. The recruited patients with AD were diagnosed according to the criteria of Hanifin and Rajka and had not been treated with any medication within 1 month. All participants provided informed consent, and the protocol and consent forms governing procedures for the study were approved by the Institutional Review Board of Seoul St. Mary’s Hospital (KC17TESI0543). Thus, 20–40 cc blood was collected from all recruited patients with AD and healthy controls.

### 2.4. Isolation of PBMCs from whole blood

Blood was collected from patients with AD and healthy controls. Peripheral blood mononuclear cells (PBMCs) were isolated using a density gradient procedure. We used density gradient solution, Histopaque^®^-1077 (Sigma Aldrich, St Louis, MO, USA). The blood sample was mixed with PBS in the ratio of 1:1. The mixed blood solution was overlayed on top of the Histopaque^®^-1077 in the ratio of 2:1 and subjected to centrifugation at 400× *g* for 35 min at room temperature. After centrifugation, the upper opaque layer containing PBMCs was aspirated out carefully and transferred into sterile conical centrifuge tubes. The suspension of PBMC was washed with PBS at 250× *g* for 10 min twice to wash off any remaining platelets. The cells were counted using Trypan blue staining. PBMCs were used immediately or frozen stored at liquid nitrogen.

### 2.5. Fluorescence-Activated Cell Sorting

PBMCs were washed twice, pooled, and resuspended to 1.0–2.0 × 10^6^ cells/mL in RPMI 1640 (Gibco, Carlsbad, CA, USA) media. Total PBMCs were incubated with CD3 Alexa fluor^®^ 405-conjugated antibody (FAB9929V-100UG; R&D system, Minneapolis, MN, USA), CD45RO (sc-1183; Santa Cruz, Dallas, TX, USA), purified anti-human/mouse CLA antibody (321302; Bio legend, San Diego, CA, USA), and anti-human H4 histamine receptor (AHR-004; Alomone Labs, Jerusalem, Israel) for 1 h at 4 °C. After washing, cells were incubated with Alexa fluor^®^ 488 goat anti-mouse IgG2a (A21131; Thermo Fisher), goat anti-rat IgG+IgM H&L (Allophycocyanin) preabsorbed (ab130798; Abcam, Cambridge, UK), and goat anti-rabbit IgG (H+L) secondary antibody with PE-cyanine 5.5 (L42018; Invitrogen, Carlsbad, CA, USA) in the dark for 30 min at 4 °C. Labeled cells were washed and filtered through a 35-μm mesh filter tube with strainer cap (352235; BD Falcon, Bedford, MA, USA). Samples were measured by FACS Aria III (BD Biosciences, CA, USA). Table 1 shows the antibodies used in this study.

### 2.6. Immunofluorescence for PBMC

CD45RO^+^CLA^+^H4R^+^ T cells were isolated from PBMC of patients with AD and healthy controls. The cells were cultured in TexMACS medium (Miltenyi Biotec, Bergisch Gladbach, Germany) containing 1% penicillin/streptomycin (Gibco) for 24 h. To activate the H4R, the cells were stimulated with or without 4MH at 10 μM (2342; TOCRIS, Bristol, UK) for 24 h. After stimulation, cells were plated onto fibronectin-coated coverslips (SPL Life Sciences, Pocheon, Korea) and allowed to adhere at 37 °C for 1 h. Following media suction, cells were fixed in 4% paraformaldehyde for 20 min at room temperature. Cells were then permeabilized with 0.1% Triton X-100 (Sigma-Aldrich) in 1X PBS for 2 min and then blocked with 1% BSA in 1X PBS for 1 h. After blocking, slides were incubated with polyclonal IgG goat antibody specific to human IL-31 (PA5-47405; Thermo Fisher) and polyclonal rabbit antibody specific to human H4R (SP4670P; OriGene Technologies) overnight at 4 °C. After rinsing with PBS, the slides were incubated with Alexa Fluor^®^ 594 chicken anti-rabbit IgG (A-21442; Thermo Fisher) and Alexa Fluor^®^ 488 donkey anti-goat IgG (A-110555; Thermo Fisher) for 2 h in the dark at room temperature. Following washes with PBS, cover slips were mounted with Vectashield Antifade Mounting Medium (Vector Laboratories) containing DAPI, to counterstain cellular nuclei. Immunofluorescence images were acquired using a fluorescence microscope (Axiovert 200, Zeiss).

### 2.7. Enzyme-Linked Immunosorbent Assay for IL-31

Isolated CD45RO^+^CLA^+^H4R^+^ T cells and CD45RO^+^CLA^+^H4R^−^ cells were seeded at a density of 1.5 × 10^4^ cells/well in 48 well plates and cultured in TexMACS medium (Miltenyi Biotec) containing 1% penicillin/streptomycin (Gibco) for 24 h. To compare the production of IL-31 between H4R-activated cells and non-activated cells, the cells were stimulated with 4MH at 10 μM (2342; TOCRIS) for 24 h [11,13]. The amount of IL-31 in culture supernatant was measured using an IL-31 enzyme-linked immunosorbent assay kit (KO331233; KOMA Biotech, Seoul, Korea) following the manufacturer’s instructions.

### 2.8. Statistical Analysis

Statistical analysis was performed with GraphPad Prism 5 software (San Diego, CA, USA). IL-31 production differences between control group and AD group were analyzed by paired t-test. All data were expressed as mean ± standard error of the mean (SEM). The level of significance was a *p* value less than 0.05 (*, *p* < 0.05; **, *p* < 0.01; and ***, *p* < 0.001).

## 3. Results

### 3.1. Co-Localization of H4R and IL-31 in Lesional AD Skin

**Figure 1:**
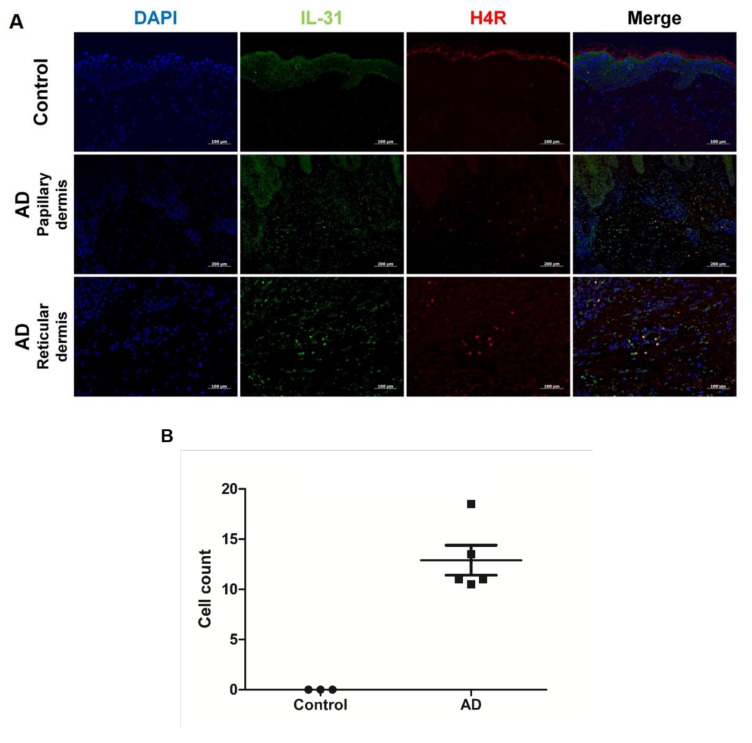
Co-localization of IL-31 and H4R in AD and healthy controls. (**A**) Overlay (yellow) of IL-31^+^ (green) and H4R^+^ (red) in AD samples and healthy controls. DAPI (blue) was used to counterstain nuclei. Original magnification × 200, scale bar = 100 μm for control and reticular dermis of AD. Original magnification × 100, scale bar = 200 μm for papillary dermis of AD. (**B**) IL-31^+^ and H4R^+^ co-localized cell counts in healthy controls and patients with AD. High-power field, × 200. Error bars indicate SEMs.

Table 2 shows demographic findings of the patients. The control group were all adult and had no prior history of allergic disease such as allergic asthma, conjunctivitis, or rhinitis and atopic dermatitis. Immunofluorescence showed colocalization of H4R and IL-31 in lesional AD skin but not in normal skin of healthy controls (Figure 1A). Co-localized cells were found almost exclusively in the dermis and around vessels. The mean number of co-localized cells in high power fields (×200) was 12.9 ± 3.3 in AD samples and 0.0 ± 0.0 in healthy control samples (Figure 1B).

### 3.2. Characteristics of AD Patients

Eight patients with AD and eight healthy controls were recruited to identify a subset of cells co-expressing H4R and IL-31. Of the eight patients with AD, five were male and three were female, and the average age was 25.6 ± 6.6 years (range 19 to 36). Healthy controls were six males and two females, with an average age of 28.0 ± 3.7 (range 24–36) and no prior history of AD or other allergic diseases such as allergic rhinitis, conjunctivitis, and asthma. In patients with AD, laboratory test results showed a proportion of eosinophils of 8.22 ± 3.24%, of IgE of 4843.9 ± 4236.3 IU/mL (range 1284 to 13,600), and of eosinophil cationic protein of 84.6 ± 44.5 mg/mL (range 26.7 to 122). Scoring atopic dermatitis index (SCORAD) was measured to be 50.0 ± 14.0 (Table 3). Of the eight patients with AD, four patients had onset in their childhoods, two patients in their adolescents, and two patients had onset as adults.

### 3.3. Comparison of CD45RO^+^CLA^+^H4R^+^ T Cells in Patients with AD and Healthy Controls

**Figure 2:**
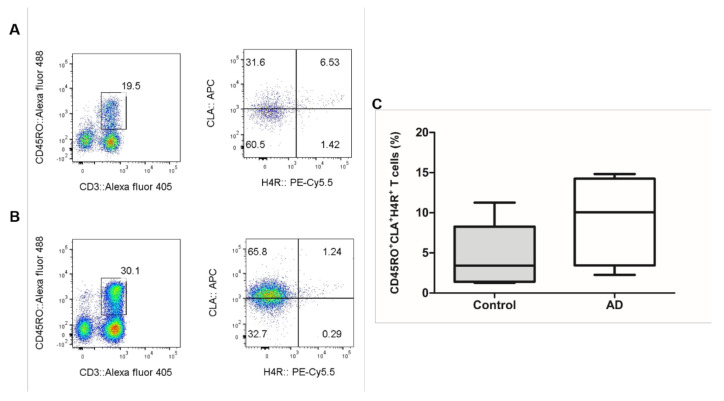
Result of CD45RO^+^CLA^+^H4R^+^ T cells in AD and healthy controls. The FACS results of CD45RO^+^CLA^+^H4R^+^ T cells in (**A**) healthy controls and (**B**) patients with AD. (**C**) Proportion of CLA^+^H4R^+^ T cells in CD3^+^CD45RO^+^ T cells; 9.1 ± 5.6% in patients with AD and 4.8 ± 3.8% in healthy control. AD, atopic dermatitis. FACS, fluorescence-activated cell sorting.

**Figure 3:**
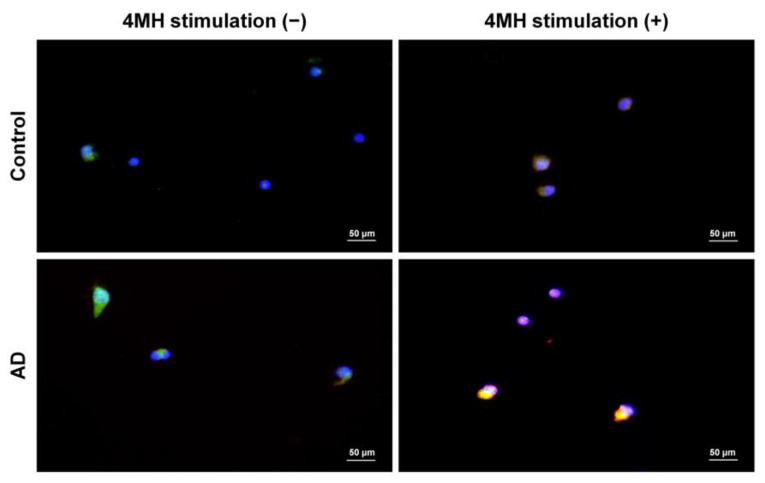
Simultaneous expression of IL-31 and H4R in sorted CD45RO^+^CLA^+^H4R^+^ T cells of AD patients with 4-methylhistamine (4MH) stimulation. Isolated CD45RO^+^CLA^+^H4R^+^ T cells from healthy controls and AD samples were cultured and stimulated with or without 4MH at 10 μM. Immunofluorescence images of IL-31 (green) and H4R (red) in sorted CD45RO^+^CLA^+^H4R^+^ T cells were observed with original magnification × 400, scale bar = 50 μm.

The proportion of CLA^+^H4R^+^ T cells in CD3^+^CD45RO^+^ T lymphocytes was 9.1 ± 5.6% in patients with AD and 4.8 ± 3.8% in healthy controls (Figure 2). To investigate if IL-31 and H4R were simultaneously expressed in sorted CD45RO^+^CLA^+^H4R^+^ T cells, IF studies were performed in samples from healthy controls and from patients with AD. The co-expression of H4R and IL-31 was observed in the AD but not the healthy control group. After 4MH stimulation, simultaneous expression of IL-31 and H4R increased in the AD group, but this change was not observed in the healthy control group (Figure 3).

**Figure 4:**
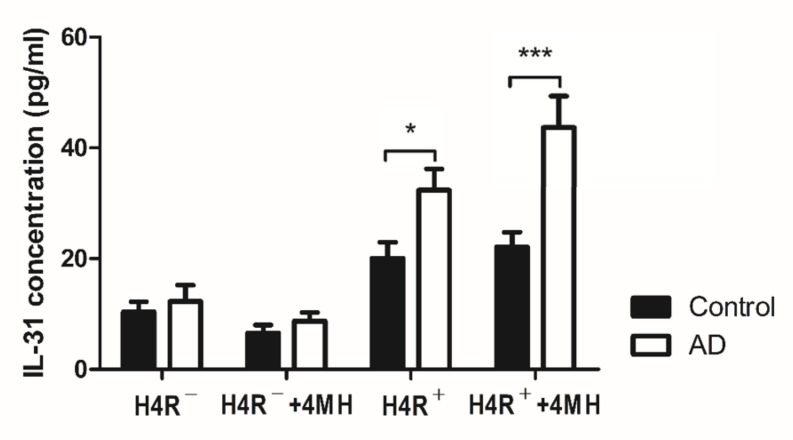
The production of IL-31 in CD45RO^+^CLA^+^H4R^+^ T cells and CD45RO^+^CLA^+^H4R^−^ T cells. CD45RO^+^CLA^+^H4R^−^ T cells and CD45RO^+^CLA^+^H4R^+^ T cells were isolated from blood samples acquired from healthy controls and patients with AD. These sorted cells were cultured at a density of 1.5 × 10^4^ cells/well and stimulated with or without 4MH at 10 μM. After stimulation for 24 h, supernatant harvested from cell medium was evaluated by ELISA for level of IL-31. * *p* < 0.05, *** *p* < 0.001.

ELISA was performed on culture supernatants of CD45RO^+^CLA^+^H4R^+^ T cells from healthy controls and of patients with AD to compare IL-31 production. As shown in Figure 4, production of IL-31 in CD45RO^+^CLA^+^H4R^+^ T cells of the AD group increased from 32.4 ± 13.3 pg/mL to 47.5 ± 18.7 pg/mL with 4MH stimulation after 24 h. However, production of IL-31 in CD45RO^+^CLA^+^H4R^+^ T cells of the control group was 20.1 ± 10.6 pg/mL without 4MH stimulation and 22.1 ± 9.3 pg/mL with stimulation. CD45RO^+^CLA^+^H4R^+^ T cells of the AD group showed more IL-31 production compared with the control group (*p* = 0.044), and this gap was wider after 4MH treatment (*p* < 0.001). The production of IL-31 in CD45RO^+^CLA^+^H4R^−^ T cells without 4MH stimulation was 10.4 ± 6.8 pg/mL in healthy controls and 12.3 ± 10.2 pg/mL in patients with AD. The production of IL-31 in CD45RO^+^CLA^+^H4R^−^ T cells was not changed significantly by 4MH stimulation in either group (Figure 4).

## 4. Discussion

IL-31 has a role in pruritus and in pathogenesis of AD [4,14]. IL-4, the Th2 cytokine, is an important factor in the expression of IL-31, but the mechanism by which IL-4 increases IL-31 expression is not clear [2,6]. Gutzmer et al. reported that H4R is upregulated on Th2 cells and by IL-4 [11]. Thus, upregulated H4R by IL-4 might be a link between IL-and IL-31.

Expression of IL-31 is reported to require a calcium signal. Park et al. found that the IL-31 promoter contains a positive regulatory region that mediates calcium- and IL-4-dependent induction of the IL-31 gene [15]. The study demonstrated that a change to an open chromatin conformation occurs in this region after stimulation with calcium and IL-4. H4R is expressed in Th2 cells, and the main signaling pathway of H4R increases intracellular Ca^2+^ [10,11]. Those previous studies support our results that H4R on CD45RO^+^CLA^+^H4R^+^ T cells induced IL-31. Although histamine-1-receptor (H1R) can also induce Ca^2+^ influx into T cells, it is more highly expressed on Th1 than Th2 cells, and histamine is known to trigger H1R to induce the Th1 response [10,16]. AD is not alleviated by oral administration of H1R antagonists [12]. This may be because AD is a typical Th2 cell-related disease, and H4R is more important than H1R in IL-31 production.

The H4R is linked to many functional inflammatory responses and upregulated by Th2 cells. Actually, H4 agonists upregulates the Th2-associated and itch-inducing IL-31 and contributes Th2 polarization which is major pathway of itching in AD [11]. H4R inhibitors and antagonists have been tested for managing pruritus and inflammatory skin lesions including AD [17,18,19,20,21,22]. Among these studies, a clinical trial of the H4R antagonist ZPL-3893787 in AD patients proved effectiveness through significantly higher reduction of eczema area severity index, and SCORAD compared with placebo [17]. The mechanisms of the H4R antagonist on AD are related to the function of H4R, which induces upregulation of IL-31 and production of TSLP, and promotes Th2 polarization and proliferation of keratinocytes [11,23,24,25,26]. However, the mode of action by which H4R antagonism blocks pruritus has not been clear yet [26]. In addition, few studies have confirmed the function of H4R in the blood and skin tissues of AD patients.

Our results showed that CD45RO^+^CLA^+^H4R^+^ T cells are an important source of IL-31 production in patients with AD. Although a proportion of this subset did not increase in the AD group compared with healthy controls, IL-31 production was significantly increased without H4R stimulation in patients with AD. In order to investigate whether IL-31 production increased after H4R stimulation, 4MH, an H4R agonist, was treated [11,13]. In the AD group, IL-31 production of this subset increased 46% in response to 4MH compared with the control group that did not respond to 4MH. According to a previous report, when CD4^+^ T cells or CD45RA^+^CD4^+^ naïve T cells derived from healthy donor are polarized into Th2 cells with IL-4 and neutralizing anti–IL-12 antibody, treatment with 4MH alone does not increase IL-31 mRNA expression, but SEB plus 4MH increases IL-31 mRNA expression [11]. Similarly, in our results, CD45RO^+^CLA^+^H4R^+^ T cells from healthy donors did not increase IL-31 production after treatment with 4MH alone. However, CD45RO^+^CLA^+^H4R^+^ T cells from patients with AD increased IL-31 production after treatment with 4MH alone. This result suggests that H4R is activated to produce IL-31 in patients with AD. It also provides indirect evidence that AD developed not only by immunologic factors, but also by environmental factors such as SEB. In CD45RO^+^CLA^+^H4R^-^ T cells, IL-31 production was significantly less than in CD45RO^+^CLA^+^H4R^+^ T cells in both groups, and production did not increase with 4MH. This result suggests that CD45RO^+^CLA^+^H4R^+^ T cells are the main source of IL-31.

AD shows heterogenous clinical findings and has various subtypes. Factors associated with distinct AD subtypes/phenotypes include immunoglobulin E level, age, disease chronicity, ethnicity, and barrier protein dysfunction [27]. For this reason, treating all subtypes of AD with the same drug is difficult. For example, the antagonizing IL-4/IL-13 agent dupilumab achieves clear or almost clear skin in only 35%–40% of AD patients, and additional treatment options are needed [27,28,29,30]. Therefore, there is need for development of a personalized medicine approach tailored to better treat AD subsets [27]. Currently, new agents for AD such as H4R antagonist [17], Janus kinase (JAK) inhibitors [31], JAK-spleen tyrosine kinase inhibitor [32], anti-OX40 antibody [33] and IL-22 monoclonal antibody [34] are being developed. In our study, although the number of patients was small, the observed IL-31 and H4R co-localized cells in the skin tissues were obtained from patients of various ages ranging from a child aged five to an adult aged 65. In addition, activated CD45RO^+^CLA^+^H4R^+^ T cells were observed in blood samples from childhood-, adolescent- and adult-onset AD patients. Investigating the action mechanism of IL-31 and H4R and discovering the CD45RO^+^CLA^+^H4R^+^ T cell subset will be the basis for future personalized and tailored treatment for patients with AD.

The main limitation of this study was the small numbers of patients and controls. Moreover, all the patients who were enrolled in this study were Korean. Although Korean patients with AD shows higher T_H_17 axis and lower T_H_1 marker than European patients, T_H_2 markers such as IL-4, 13 and 31 were similar [35]. Pediatric AD also shows increased T_H_17 axis and IL-22, but T_H_2 is increased like other subtypes of AD. Likewise, T_H_2 activation is observed in the pathomechanism of overall AD, including both chronic and acute AD, and both intrinsic and extrinsic AD [35]. Therefore, treatment targeting IL-31 or IL-31 receptor is applied to all AD phenotypes [35]. From this point of view, although our study was conducted on a limited group of patients, it could also be applied to other phenotypes of AD. Nevertheless, the number of patients was too small to include various subtypes of AD, and it is necessary to investigate more in detail whether CD45RO^+^CLA^+^H4R^+^ T cell subset is involved in each subtype of AD in the future for precision medicine. Although the number of patients was small, since this T cell subset was observed in AD at various ages and at various onset periods, we think it is worth further research.

In summary, CD45RO^+^CLA^+^H4R^+^ T cells are an important source of IL-31 in AD, and this subset or H4R may be a treatment target for AD.

## Figures and Tables

**Table 1:** Primary and secondary antibody used for flow cytometry.

Target	Primary Antibody		Secondary Antibody		
	Clonality	Host	Fluorophore	Reactivity	Host
CD3	Monoclonal	Human	Alexa fluor^®^ 405	Conjugated	
CD45RO	Monoclonal	Mouse	Alexa fluor^®^ 488	Mouse	Goat
CLA	Monoclonal	Rat	Allophycocyanin	Rat	Goat
H4R	Polyclonal	Rabbit	PE-cyanine 5.5	Rabbit	Goat

**Table 2:** Demographic findings of AD patients (skin biopsy).

Case ID	Age	Sex	IgE (IU/mL)	Biopsy Site
1	5	M	NA	Back
2	23	F	NA	Back
3	24	F	2280	Thigh
4	37	F	2153	Lowe leg
5	62	M	3412	Face
Total	30.2 ± 21.1		2615 ± 693	

NA, not assessed.

**Table 3:** Demographic findings of AD patients (blood samples).

Case ID	Age	Sex	Eosinophils (%)	IgE (IU/mL)	ECP (ng/mL)	SCORAD
AD1	20	M	8.2	NA	NA	54.6
AD2	19	M	2.2	1284	26.7	39.5
AD3	36	F	10.1	2346	46.5	35.0
AD4	20	M	7.5	5369	114	49.1
AD5	35	F	6.9	1968	122	35.9
AD6	26	M	10.9	13,600	NA	56.5
AD7	24	F	7.0	3349	114	51.5
AD8	25	M	13.0	5991	NA	77.6
Total	25.6 ± 6.6		8.2 ± 3.2	4843.9 ± 4236.3	84.6 ± 44.5	50.0 ± 14.0

ECP, eosinophil cationic protein; NA, not assessed; SCORAD, scoring atopic dermatitis index.

## Data Availability

The data presented in this study are available in the article.

## Abbreviations

4MH	4-methylhistamine dihydrochloride
AD	atopic dermatitis
BSA	bovine serum albumin
CLA	cutaneous lymphocyte-associated antigen
FACS	fluorescence-activated cell sorting
H1R	histamine-1-receptor
H4R	histamine-4-receptor
IF	immunofluorescence
IL	interleukin
JAK	Janus kinase
PBMC	peripheral blood mononuclear cell
SEB	staphylococcal enterotoxin B
TSLP	thymic stromal lymphopoietin

## References

[1] Dillon S.R., Sprecher C., Hammond A., Bilsborough J., Rosenfeld-Franklin M., Presnell S.R., Haugen H.S., Maurer M., Harder B., Johnston J. (2004). Interleukin 31, a cytokine produced by activated T cells, induces dermatitis in mice. Nat. Immunol..

[2] Bagci I.S., Ruzicka T. (2018). IL-31: A new key player in dermatology and beyond. J. Allergy Clin. Immunol..

[3] Ezzat M.H., Hasan Z.E., Shaheen K.Y. (2011). Serum measurement of interleukin-31 (IL-31) in paediatric atopic dermatitis: Elevated levels correlate with severity scoring. J. Eur. Acad. Dermatol. Venereol..

[4] Ruzicka T., Hanifin J.M., Furue M., Pulka G., Mlynarczyk I., Wollenberg A., Galus R., Etoh T., Mihara R., Yoshida H. (2017). Anti-Interleukin-31 Receptor A Antibody for Atopic Dermatitis. N. Engl. J. Med..

[5] Bilsborough J., Leung D.Y., Maurer M., Howell M., Boguniewicz M., Yao L., Storey H., LeCiel C., Harder B., Gross J.A. (2006). IL-31 is associated with cutaneous lymphocyte antigen-positive skin homing T cells in patients with atopic dermatitis. J. Allergy Clin. Immunol..

[6] Stott B., Lavender P., Lehmann S., Pennino D., Durham S., Schmidt-Weber C.B. (2013). Human IL-31 is induced by IL-4 and promotes TH2-driven inflammation. J. Allergy Clin. Immunol..

[7] Cevikbas F., Wang X., Akiyama T., Kempkes C., Savinko T., Antal A., Kukova G., Buhl T., Ikoma A., Buddenkotte J. (2014). A sensory neuron-expressed IL-31 receptor mediates T helper cell-dependent itch: Involvement of TRPV1 and TRPA1. J. Allergy Clin. Immunol..

[8] Maier E., Werner D., Duschl A., Bohle B., Horejs-Hoeck J. (2014). Human Th2 but not Th9 cells release IL-31 in a STAT6/NF-kappaB-dependent way. J. Immunol..

[9] Cornelissen C., Brans R., Czaja K., Skazik C., Marquardt Y., Zwadlo-Klarwasser G., Kim A., Bickers D.R., Luscher-Firzlaff J., Luscher B. (2011). Ultraviolet B radiation and reactive oxygen species modulate interleukin-31 expression in T lymphocytes, monocytes and dendritic cells. Br. J. Dermatol..

[10] Thurmond R.L., Gelfand E.W., Dunford P.J. (2008). The role of histamine H1 and H4 receptors in allergic inflammation: The search for new antihistamines. Nat. Rev. Drug. Discov..

[11] Gutzmer R., Mommert S., Gschwandtner M., Zwingmann K., Stark H., Werfel T. (2009). The histamine H4 receptor is functionally expressed on T(H)2 cells. J. Allergy Clin. Immunol..

[12] Ohsawa Y., Hirasawa N. (2014). The role of histamine H1 and H4 receptors in AD. Allergol. Int..

[13] Raap U., Gehring M., Kleiner S., Rudrich U., Eiz-Vesper B., Haas H., Kapp A., Gibbs B.F. (2017). Human basophils are a source of—and are differentially activated by—IL-31. Clin. Exp. Allergy.

[14] Feld M., Garcia R., Buddenkotte J., Katayama S., Lewis K., Muirhead G., Hevezi P., Plesser K., Schrumpf H., Krjutskov K. (2016). The pruritus- and TH2-associated cytokine IL-31 promotes growth of sensory nerves. J. Allergy Clin. Immunol..

[15] Park K., Park J.H., Yang W.J., Lee J.J., Song M.J., Kim H.P. (2012). Transcriptional activation of the IL31 gene by NFAT and STAT6. J. Leukoc. Biol..

[16] Jutel M., Watanabe T., Akdis M., Blaser K., Akdis C.A. (2002). Immune regulation by histamine. Curr. Opin. Immunol..

[17] Werfel T., Layton G., Yeadon M., Whitlock L., Osterloh I., Jimenez P., Liu W., Lynch V., Asher A., Tsianakas A. (2019). Efficacy and safety of the histamine H4 receptor antagonist ZPL-3893787 in patients with atopic dermatitis. J. Allergy Clin. Immunol..

[18] Rossbach K., Wendorff S., Sander K., Stark H., Gutzmer R., Werfel T., Kietzmann M., Baumer W. (2009). Histamine H4 receptor antagonism reduces hapten-induced scratching behaviour but not inflammation. Exp. Dermatol..

[19] Seike M., Furuya K., Omura M., Hamada-Watanabe K., Matsushita A., Ohtsu H. (2010). Histamine H(4) receptor antagonist ameliorates chronic allergic contact dermatitis induced by repeated challenge. Allergy.

[20] Dunford P.J., Williams K.N., Desai P.J., Karlsson L., McQueen D., Thurmond R.L. (2007). Histamine H4 receptor antagonists are superior to traditional antihistamines in the attenuation of experimental pruritus. J. Allergy Clin. Immunol..

[21] Murata Y., Song M., Kikuchi H., Hisamichi K., Xu X.L., Greenspan A., Kato M., Chiou C.F., Kato T., Guzzo C. (2015). Phase 2a, randomized, double-blind, placebo-controlled, multicenter, parallel-group study of a H4 R-antagonist (JNJ-39758979) in Japanese adults with moderate atopic dermatitis. J. Dermatol..

[22] Kollmeier A., Francke K., Chen B., Dunford P.J., Greenspan A.J., Xia Y., Xu X.L., Zhou B., Thurmond R.L. (2014). The histamine H(4) receptor antagonist, JNJ 39758979, is effective in reducing histamine-induced pruritus in a randomized clinical study in healthy subjects. J. Pharmacol. Exp. Ther..

[23] Dijkstra D., Stark H., Chazot P.L., Shenton F.C., Leurs R., Werfel T., Gutzmer R. (2008). Human inflammatory dendritic epidermal cells express a functional histamine H4 receptor. J. Investig. Dermatol..

[24] Gutzmer R., Diestel C., Mommert S., Kother B., Stark H., Wittmann M., Werfel T. (2005). Histamine H4 receptor stimulation suppresses IL-12p70 production and mediates chemotaxis in human monocyte-derived dendritic cells. J. Immunol..

[25] Glatzer F., Gschwandtner M., Ehling S., Rossbach K., Janik K., Klos A., Baumer W., Kietzmann M., Werfel T., Gutzmer R. (2013). Histamine induces proliferation in keratinocytes from patients with atopic dermatitis through the histamine 4 receptor. J. Allergy Clin. Immunol..

[26] Schaper K., Rossbach K., Kother B., Stark H., Kietzmann M., Werfel T., Gutzmer R. (2016). Stimulation of the histamine 4 receptor upregulates thymic stromal lymphopoietin (TSLP) in human and murine keratinocytes. Pharmacol. Res..

[27] Renert-Yuval Y., Guttman-Yassky E. (2020). New treatments for atopic dermatitis targeting beyond IL-4/IL-13 cytokines. Ann. Allergy Asthma. Immunol..

[28] Thaçi D., Simpson E.L., Beck L.A., Bieber T., Blauvelt A., Papp K., Soong W., Worm M., Szepietowski J.C., Sofen H. (2016). Efficacy and safety of dupilumab in adults with moderate-to-severe atopic dermatitis inadequately controlled by topical treatments: A randomised, placebo-controlled, dose-ranging phase 2b trial. Lancet.

[29] Simpson E.L., Bieber T., Guttman-Yassky E., Beck L.A., Blauvelt A., Cork M.J., Silverberg J.I., Deleuran M., Kataoka Y., Lacour J.P. (2016). Two Phase 3 Trials of Dupilumab versus Placebo in Atopic Dermatitis. N. Engl. J. Med..

[30] Blauvelt A., de Bruin-Weller M., Gooderham M., Cather J.C., Weisman J., Pariser D., Simpson E.L., Papp K.A., Hong H.C.-H., Rubel D. (2017). Long-term management of moderate-to-severe atopic dermatitis with dupilumab and concomitant topical corticosteroids (LIBERTY AD CHRONOS): A 1-year, randomised, double-blinded, placebo-controlled, phase 3 trial. Lancet.

[31] Guttman-Yassky E., Silverberg J.I., Nemoto O., Forman S.B., Wilke A., Prescilla R., de la Pena A., Nunes F.P., Janes J., Gamalo M. (2019). Baricitinib in adult patients with moderate-to-severe atopic dermatitis: A phase 2 parallel, double-blinded, randomized placebo-controlled multiple-dose study. J. Am. Acad. Dermatol..

[32] Bissonnette R., Maari C., Forman S., Bhatia N., Lee M., Fowler J., Tyring S., Pariser D., Sofen H., Dhawan S. (2019). The oral Janus kinase/spleen tyrosine kinase inhibitor ASN002 demonstrates efficacy and improves associated systemic inflammation in patients with moderate-to-severe atopic dermatitis: Results from a randomized double-blind placebo-controlled study. Br. J. Dermatol..

[33] Guttman-Yassky E., Pavel A.B., Zhou L., Estrada Y.D., Zhang N., Xu H., Peng X., Wen H.C., Govas P., Gudi G. (2019). GBR 830, an anti-OX40, improves skin gene signatures and clinical scores in patients with atopic dermatitis. J. Allergy Clin. Immunol..

[34] Guttman-Yassky E., Brunner P.M., Neumann A.U., Khattri S., Pavel A.B., Malik K., Singer G.K., Baum D., Gilleaudeau P., Sullivan-Whalen M. (2018). Efficacy and safety of fezakinumab (an IL-22 monoclonal antibody) in adults with moderate-to-severe atopic dermatitis inadequately controlled by conventional treatments: A randomized, double-blind, phase 2a trial. J. Am. Acad. Dermatol..

[35] Czarnowicki T., He H., Krueger J.G., Guttman-Yassky E. (2019). Atopic dermatitis endotypes and implications for targeted therapeutics. J. Allergy Clin. Immunol..

